# Supplementary material for: Ethical and methodological reflections: Digital storytelling of self-care with students during the COVID-19 pandemic at a South African University
Source: PLOS Glob Public Health. 2023 Jun 14;3(6):e0001682. doi: 10.1371/journal.pgph.0001682 (PMC10266614; doi:10.1371/journal.pgph.0001682)
Supplement: Attachment — Submitted filename: Response to Reviewers.docx [file pgph.0001682.s001.docx]

**RESPONSE TO REVIEWERS: PGPH-D-23-00030**

**Comments from Reviewer #1:**

This is an interesting paper on digital story telling. My main comment is that the authors must include more literature on DST in the background e.g. why was DST developed, what were the intended goals of DST, what challenges have been experienced elsewhere etc DiFulvio GT, (2016), Fiddian-Green A, (2019), de Jager A, (2017), Nyirenda (2022) and Rieger (2018) are some of the references.

RESPONSE: Thank you for highlighting the gaps in the background section. We acknowledge that we may have not sufficiently incorporated enough literature on DST in the background. The background section is now amended, as follows:

A systematic review highlighted five goals of digital storytelling in public health interventions and research: a) digital stories as ‘counter-narratives’; b) knowledge translation; c) preservation of cultural heritage; d) community development; and e) participatory research with marginalized groups (26). While digital storytelling shows promise for public health research, there are also challenges that must be navigated (21). Specific challenges include the quality of digital stories produced (27); the potential of researchers shaping participants’ digital stories and lack of participants’ confidentiality (21); and concerns of stigma if digital stories are shared with the public (24). Despite the reported challenges, digital storytelling could be modified to address the ethical and logistical challenges of working with vulnerable groups (25). Given the growing interest in digital storytelling, it warrants critical examination.

**Methodology**

Please have a statement on positionality- indicate who are the data collectors, what are their roles? Are they students or lecturers? Could their social role have an impact on data collected?

RESPONSE: Thank you for raising this important point to disclose a statement on positionality. This has been clarified in the methodology section by stating:

The first author (DG) is a post-doctoral fellow trained in qualitative data collection and speaks both isiZulu (the local language of the participants) and English fluently. DG was responsible for data collection. MNS is a full professor and co-investigator in the study, whose responsibility included checking for the accuracy and quality of data. Both DG and MNS were employed at the university; however, they were not involved in teaching the target participants and thus indirectly linked to the participants.

Additionally, in the study information sheet, target participants were informed that:

Accepting or declining to take part in the study will not impact their academic marks or jeopardize their registration status within the university or target them in any way. Participants will have the right to withdraw their participation from the project at any time.

How were participants compensated for their time or why were they not compensated?

RESPONSE: Eligible participants were included voluntarily and were not compensated. This was clearly stated in the informed consent process.

How did the authors consent for e.g. family members or friends featured in DST?

RESPONSE: Participants were requested to ensure that they obtain permission for family members or friends featured in DST. However, none of the participants’ digital stories featured family members or friends except the participants themselves.

How did they plan to support individuals who demonstrated potential for self-harm as part of self-care?

RESPONSE: We had planned to refer individuals who demonstrated potential for self-harm to the available university’s psychosocial support services. However, none of the participants demonstrated potential for self-harm and thus no referrals were made.

How did they determine the sample size?

RESPONSE: Thank you. We now state that a purposive sample of between 20 to 30 students was selected to participate in the study until saturation was reached as evidenced by similar responses being made by participants. Criteria for selecting the participants were as follows: first-year students who were registered for the first time in higher education in the academic year 2022. Returning students were excluded from participation in the study.

How many invitations were sent?

RESPONSE: Thank you. We have now clarified that an open invitation to participate in the study was sent to all individuals who met the inclusion criteria through student notices and student email accounts. The invitation contained a Microsoft Teams link to access the study information letter and the informed consent form. The invitation was sent out twice in March 2022.

How many refusals & how many people did not respond?

RESPONSE: It was not possible to calculate the number of refusals because only consenting individuals completed the online informed consent form. We have now clarified in the manuscript that of 29 consenting individuals, only three withdrew from participation by not submitting their digital stories. The reasons for withdrawal were not captured.

Did you get more willing students than the required sample size & how was this dealt with?

RESPONSE: No, we got the required sample size as explained above.

Did they all submit the DST or were there defaulters?

RESPONSE: As previously mentioned above, of 29 consenting individuals, a total of 26 students submitted their digital stories and only three were defaulters for unknown reasons. However, as stated in the manuscript, the majority of participants did not submit digital stories by the specified deadline, reporting a lack of time due to their academic workload. It is therefore possible that the defaulters were overloaded with academic work and thus withdrew from participation. As a solution to a lack of time, participants were advised to choose any time at their convenience to work on their digital stories while allowing them the flexibility to submit their stories regardless of the specified deadline.

**Discussion**

What if the study was not done among students- where you can easily access email address? How else can you reach out to hard to reach, marginalised or minority groups in LMICs- in an emergency situation- among who do not even have emails?

RESPONSE: Thank you for raising these important questions. Based on our extensive experience in qualitative studies that involve hard-to-reach or marginalized groups who do emails, mobile telephones instead of emails are an option to reach out to those individuals.

Was there any likelihood that first year students would present self-care practices that were perceived to be socially acceptable? What if they did?

RESPONSE: Thank you for raising this important question. As stated in the manuscript, we opted to hold one-on-one sessions via MS Teams to guide participants on basic steps for creating digital stories in order to ensure the privacy and confidentiality of participants instead of running digital storytelling workshops with the participants as a group. We have now added that:

This strategy was aimed at preventing participants from presenting self-care practices that could have been perceived as socially acceptable in a group workshop.

We have also provided an additional explanation in the manuscript on the point of social desirability bias:

Furthermore, to minimize social desirability in the study, participants were informed that any answer is acceptable in terms of how they practice self-care and were encouraged to be honest.

**Other general comments**

Lines 78-80- Can authors comment whether people with disabilities e.g., blind people or socio economically poor people who not have smart phones or ability to read emails can participate in this type of DST?

RESPONSE: Thank you. This important statement has been added to the manuscript.

Line 98 -99- It is not clear what they mean by authors may present. Which authors are they referring to?

RESPONSE: Thank you for pointing out this statement, which is confusing. We have now deleted it and rephrased it as follows:

Despite the importance of digital storytelling in research, there remains a paucity of evidence on ethical and methodological challenges in applying this method.

Line 113- STIs including HIV & unplanned pregnancies…Please rephrase because it sounds like an unplanned pregnancy is an example of STI.

RESPONSE: Thank you. This has been amended.

The authors keep using First time entering students and first year students- Can they be consistent? First time entering students is ambiguous because it doesn’t say first time entering what.

RESPONSE: Thank you. Although the term ‘first-time entering student’ is widely used in South Africa to mean a registered student for an undergraduate programme who has not in the past been effectively registered in any university. We acknowledge that the term may be confusing and not used in other contexts. Therefore, we have now changed “first-time entering student” to “first-year university student”. We hope this revision provides a clearer understanding.

Line 228-230 Check the grammar & simplify the sentence.

RESPONSE: Thank you. The sentence has been corrected.

Line 405 & several other places- please use LMIC & not LMMC.

RESPONSE: Thank you. The correction has been made in the manuscript.

**Comments from Reviewer #2:**

This is a well written paper, setting out clearly the ethical and methodological challenges faced in this study. The ethical challenges encountered are very clearly explained, as are the potential solutions to the challenges. For the purposes of this paper, the authors explain that they are reflecting on the research process. I therefore understood this to be a reflective paper (that focuses on methodological challenges), and not a statistical analysis of results, hence my response to question 2 above (I don't think a statistical analysis is relevant in this case). The paper is wonderfully clear and detailed and can help other researchers who are carrying out remote data collection in a similar context.

RESPONSE: Thank you so much for acknowledging the importance of our work. We hope other researchers who carry out remote data collection would find our work useful.
